# Urban delineation through a prism of intraday commute patterns

**DOI:** 10.3389/fdata.2024.1356116

**Published:** 2024-03-05

**Authors:** Yuri Bogomolov, Alexander Belyi, Stanislav Sobolevsky

**Affiliations:** ^1^Department of Mathematics and Statistics, Faculty of Science, Masaryk University, Brno, Czech Republic; ^2^Center for Urban Science and Progress, New York University, New York, NY, United States

**Keywords:** commute patterns, suburban commute, city delineation, mobile phone data, intraday commute patterns

## Abstract

**Introduction:**

Urban mobility patterns are crucial for effective urban and transportation planning. This study investigates the dynamics of urban mobility in Brno, Czech Republic, utilizing the rich dataset provided by passive mobile phone data. Understanding these patterns is essential for optimizing infrastructure and planning strategies.

**Methods:**

We developed a methodological framework that incorporates bidirectional commute flows and integrates both urban and suburban commute networks. This comprehensive approach allows for a detailed representation of Brno's mobility landscape. By employing clustering techniques, we aimed to identify distinct mobility patterns within the city.

**Results:**

Our analysis revealed consistent structural features within Brno's mobility patterns. We identified three distinct clusters: a central business district, residential communities, and an intermediate hybrid cluster. These clusters highlight the diversity of mobility demands across different parts of the city.

**Discussion:**

The study demonstrates the significant potential of passive mobile phone data in enhancing our understanding of urban mobility patterns. The insights gained from intraday mobility data are invaluable for transportation planning decisions, allowing for the optimization of infrastructure utilization. The identification of distinct mobility patterns underscores the practical utility of our methodological advancements in informing more effective and efficient transportation planning strategies.

## 1 Introduction

City planning relies on a comprehensive understanding of commute patterns, given its substantial impact on decision-making regarding transportation infrastructure, public transport services, and urban development. To predict commute flows, current models are built upon the spatial structure of cities as a foundational framework. The widely employed approach is the four-step transportation model (McNally, 2007). Comprising four sequential steps, namely trip generation, trip distribution, mode choice, and trip assignment, this model facilitates a comprehensive understanding of urban transportation dynamics. Initially, trips are generated through the integration of population and household data. Subsequently, the model establishes linkages between trip origins and destinations by leveraging commute patterns.

The commute patterns are often modeled using variations of the gravity model (Zipf, 1946), which predicts the flow of people between two locations based on their respective masses (population, economic activity, etc.). Another alternative is the radiation model (Simini et al., 2012), which considers the characteristics of neighboring nodes. Brockmann et al. (2006) studied human mobility based on the circulation of bank notes in the United States. All these models work with static data and do not describe commute dynamics. Comprehending intraday commute patterns has broad implications for transportation planning, urban development, emergency preparedness, and public health (Gao et al., 2019).

In exploring intraday commute patterns, the research is constrained by the limited availability of hourly datasets. Previous endeavors have predominantly focused on specific mobility flows, such as bicycle commuting in Melbourne (Smith and Kauermann, 2011), taxi trips in New York City (Buchholz, 2015) and bus trips in Fortaleza, Brazil (Ponte et al., 2021). Social media check-in data is another great source of mobility data (Cho et al., 2011; Wu et al., 2014). In addition to the location, the check-in data often contains information on the user's motivation to visit a particular place. However, social media usage is inconsistent across age, online experience, and socioeconomic status (Hargittai, 2015).

The utilization of mobile phone data has proven to be successful in various aspects of understanding urban dynamics. Notably, it has been employed to study city structure based on population density (Xinyi et al., 2015), analyze mobility patterns (Yu et al., 2020; Zhang et al., 2022), investigate their relation to socioeconomic status (Xu et al., 2018, 2019), delineate urban park catchment areas (Guan et al., 2020), and examine changes in the average distance between individuals (Louail et al., 2014). Despite these achievements, the full potential of mobile phone data in comprehending intraday commute patterns remains largely untapped, highlighting the need for further research in this area.

The challenge of defining city and district boundaries has been acknowledged in previous studies (Bettencourt et al., 2010; Bettencourt, 2013). Prior attempts to define district signatures and employ them for district clustering were predominantly based on datasets from municipal requests (Wang et al., 2017), financial activities (Sobolevsky et al., 2014), and mobile call records (Ratti et al., 2010; Pei et al., 2014; Xu et al., 2017). However, these events are only generated in response to specific activities, resulting in limited coverage.

In our previous work (Bogomolov et al., 2023), we proposed an approach harnessing intraday mobility patterns data from mobile phones to establish distinct signatures for urban locations and subsequently apply them to urban zoning. Delineated city zones were spatially cohesive and had distinct commute patterns. The paper is based on the commute data in the city of Brno. However, the rapidly rising urban sprawl (Behnisch et al., 2022) requires considering suburban commute. Big urban areas attract people from other areas: about 20% of the 4.7 million employees working in New York City live outside the city (Planning, 2019), and more than 20% of Europeans commute at least 90 min daily (Worx, 2018).

This paper presents a significant advancement in our methodology, tailored to combine data across multiple commute networks. We delineate city districts based on the intraday commute patterns of people who commute to Brno from the suburban area in addition to Brno residents. The combination of intercity and suburban commute patterns provides better insights into transportation needs, which can be used for urban and transportation planning.

## 2 Materials and methods

### 2.1 Data

In the realm of mobile phone datasets, two primary types exist: active and passive. Active datasets involve records of specific actions, such as phone calls or text messages. Notably, every phone maintains communication with the mobile phone network at least two times per hour, thereby facilitating the capture of passive datasets. Based on that, passive datasets offer superior coverage for studying mobility patterns and population density. For our research, we rely on using the passive mobile phone dataset provided by a local company.

Building on our prior work (Bogomolov et al., 2023), where we relied on hourly origin-destination flow data from a mobile phone company covering one week in October 2019 and capturing movements of residents of 48 districts in Brno. It's essential to note that the initial dataset lacked information on suburban commuters. In the current study, we enhance our approach by incorporating a significantly more comprehensive origin-destination flow dataset encompassing the entire South Moravian Region. This expansion proves particularly valuable given the considerable number of commuters traveling to and from Brno.

The South Moravian Region, with a population of approximately 1,200,000 individuals, presents a marked contrast to the 380,000 inhabitants within the city of Brno. As a regional capital and a pivotal transportation hub, Brno acts as a focal point, drawing in commuters across the entire South Moravian Region. This regional attractiveness is further heightened by an extensive transportation network facilitating access to remote areas. For instance, the town of Znojmo can be reached within an hour from Brno via public transportation. Including these suburban commuting patterns in our updated dataset provides a more holistic understanding of the dynamic flows within the region. Based on the dataset, 23% of Brno commuters traveled from South Moravia, which aligns with the 20% out-of-city commuters estimates in EU cities (Worx, 2018) and New York (Planning, 2019). The dataset contains aggregated origin-destination flows for 32 thousand Brno and South Moravia residents.

Due to data sensitivity, the dataset does not contain any individual information and does not provide district-level granularity for suburban areas. However, it still showed the destination district for commuters to Brno, allowing us to aggregate all incoming and outgoing flows from the South Moravian Region. To study intraday commute patterns, we compute four vectors for every city district:

1. Incoming city hourly flow.
2. Outgoing city hourly flow.
3. Incoming suburban hourly flow.
4. Outgoing suburban hourly flow.

Each vector has 168 components (the number of hours within 1 week), where each component represents the number of people departing from or arriving at the given city district.

### 2.2 Data processing and clustering

The four vectors for each district are normalized using the total sum of all vector components and concatenated into one signature vector with 168 × 4 = 672 components. Please note that vector dimensionality does not depend on the number of commuters or trips. In our dataset, we had 3 million commute records, and they were aggregated on a single machine. However, the same approach can be applied to process billions of trip records. The node-level inflow/outflow data aggregation can be done using standard distributed computing tools. While downstream processing steps work only with condensed district signature vectors.

**Figure 1:**
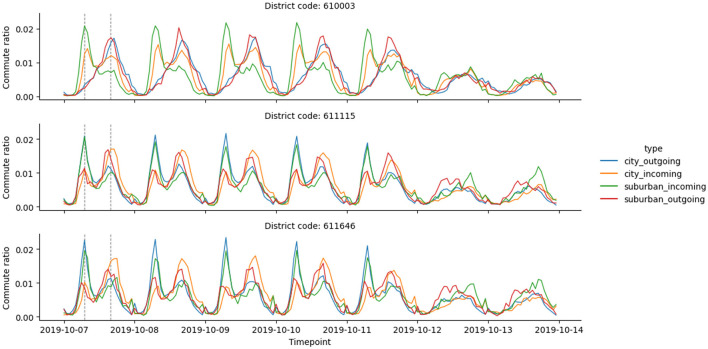
The visual depicts the hourly commute ratios (the ratio of weekly commute observed within a given hour) in three Brno districts. The first five data days illustrate weekdays, whereas the final two days depict the weekend. The vertical dashed lines emphasize surges in commuting activity, aligning with 8 am and 5 pm during weekdays.

The analysis of the resulting commute vectors revealed patterns that align with the expected daily and weekly rhythms, as seen in Figure 1. Notably, one can observe that:

- Weekends exhibit distinct patterns.
- Weekdays follow another set of patterns, while Friday commute numbers typically deviate from the other four days.
- Correlation exists between urban and suburban inflow/outflow components.

We applied PCA (Principal Component Analysis) to reduce vectors' dimensionality, reduce the impact of repeating patterns, and concentrate on the orthogonal elements. We used 7-component PCA vectors that explain 81.82% of cumulative variance in the 672-component input vectors.

Figure 1 demonstrates that some districts have consistent patterns across all four timelines. Using K-Means with three clusters (decided based on the elbow method for the within-cluster sum of squares) provides a city delineation based on the similarity of commute patterns.

### 2.3 Cluster probability visualization

Visualization methods facilitate the interpretation of complex datasets by converting raw information into visually comprehensible representations. Through graphs, charts, and maps, intricate patterns and trends within urban data become more accessible to city planners.

We wanted to highlight city districts that share traits of different clusters. To achieve that goal, we compute the cluster probability (*p*_*ij*_) for every district *i* and cluster *j* based on the distance between district vectors and cluster centers using a Gaussian kernel (Equation 1):


(1) $$p_{ij}=\frac{\exp\left(-\frac{d_{ij}^{2}}{2\sigma_{i}^{2}}\right)}{\sum_{k=1}^{3}\exp\left(-\frac{d_{ik}^{2}}{2\sigma_{i}^{2}}\right)}$$

where *d*_*ij*_ is the Euclidean distance between vector *x*_*i*_ and cluster centroids *c*_*j*_, and $\sigma_{i}^{2}$ is the minimum squared distance for each data point among its distances to all cluster centroids. The district color code is computed as the weighted sum of red, green, and blue colors, using cluster probabilities as color weights (Equation 2):


(2) $$\begin{array}{l} RGB_{i}=\left(p_{i1}\times \text{Red}\right)+\left(p_{i2}\times \text{Green}\right)+\left(p_{i3}\times \text{Blue}\right) \end{array}$$

This formula computes the mixed color by weighting each RGB component based on the normalized probabilities of each color.

## 3 Results

**Figure 2:**
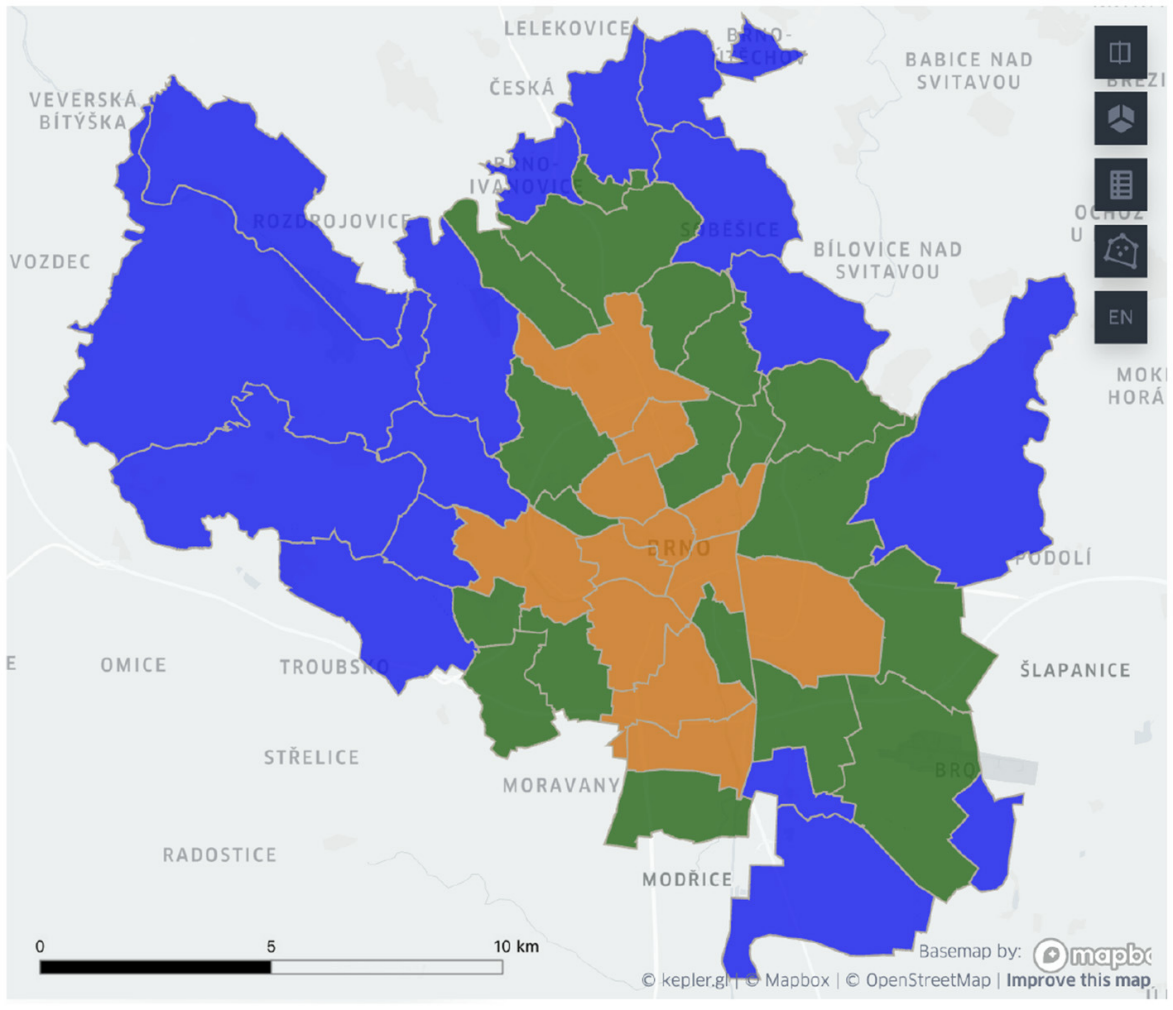
Brno delineation based on the combination of urban and suburban incoming and outgoing hourly mobile phone-based commuter patterns. Blue color represents the residential cluster, orange is used for the central cluster, and green highlights the hybrid cluster.

Figure 2 demonstrates the clustering results based on the combination of urban and suburban hourly commute timelines. The clusters are spatially cohesive, and they form structural patterns:

- The city center cluster.
- Residential communities.
- And a hybrid cluster in-between with traces of both patterns.

**Figure 3:**
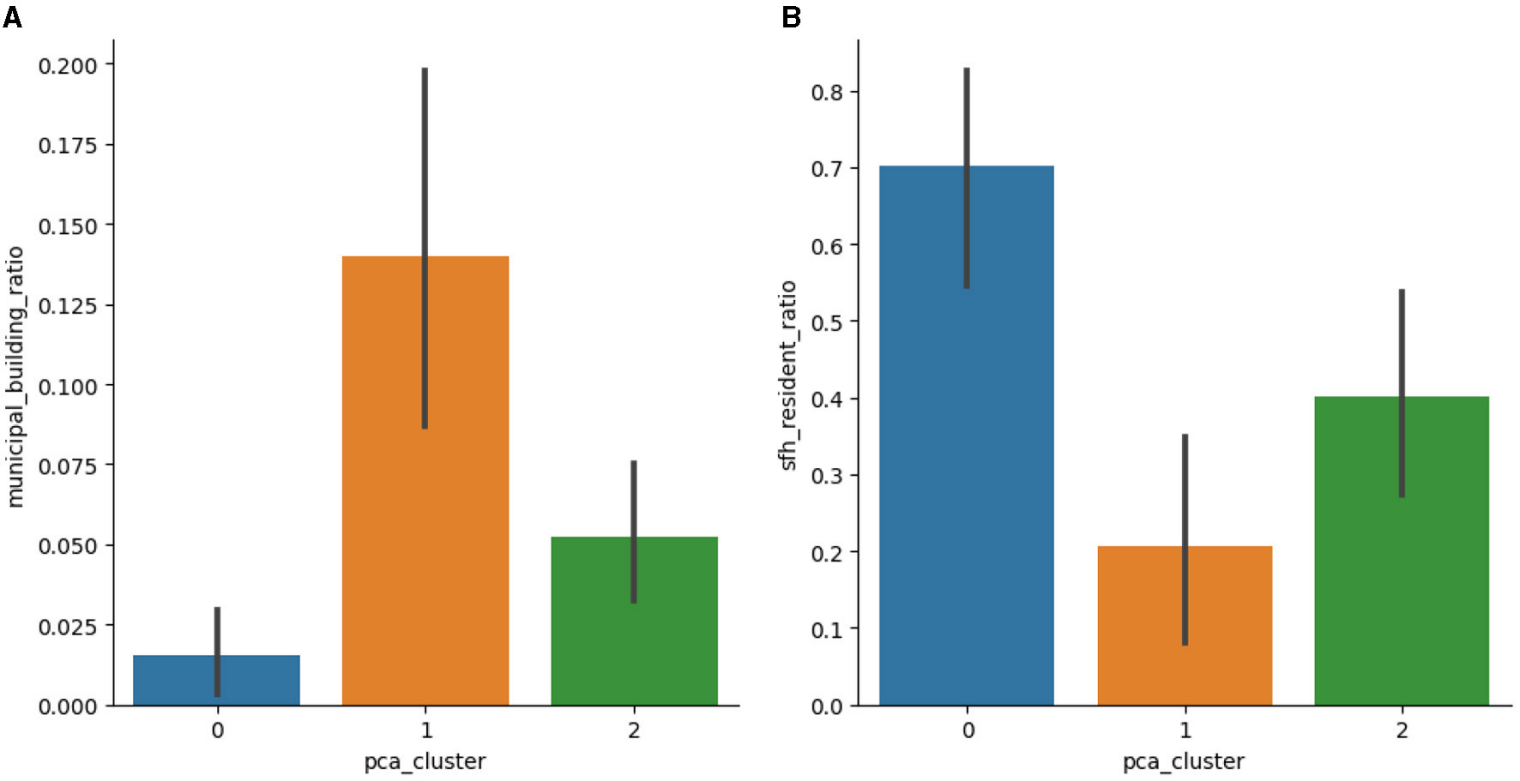
Comparison of the building and resident profiles for resulting clusters. The ratio of **(A)** municipal buildings among all district buildings and **(B)** single-family home residents among all residents within each district, including 95% confidence intervals to display the range of these percentages among various locations within the zone. **(A)** Municipal building ratio. **(B)** Single family home resident ratio.

The delineated urban zones have distinctive socio-economic profiles: the municipal home ratio and the ratio of residents living in single-family homes between the clusters are different by at least 200% between the clusters. Refer to Figure 3 for more details.

Given the same structure of the clusters, we performed a quantitative comparison of clustering results with the previous approach (Bogomolov et al., 2023). We received a Surprisingly, 93.75% of districts belong to the same cluster, while previously considered commute timeline signature vector components correspond to only 25% of the proposed signature vector. The new clusters have the same or lower average variance values of census-based urban metrics (the ratio of municipal buildings and single-family home residents), representing a higher district similarity within clusters.

**Figure 4:**
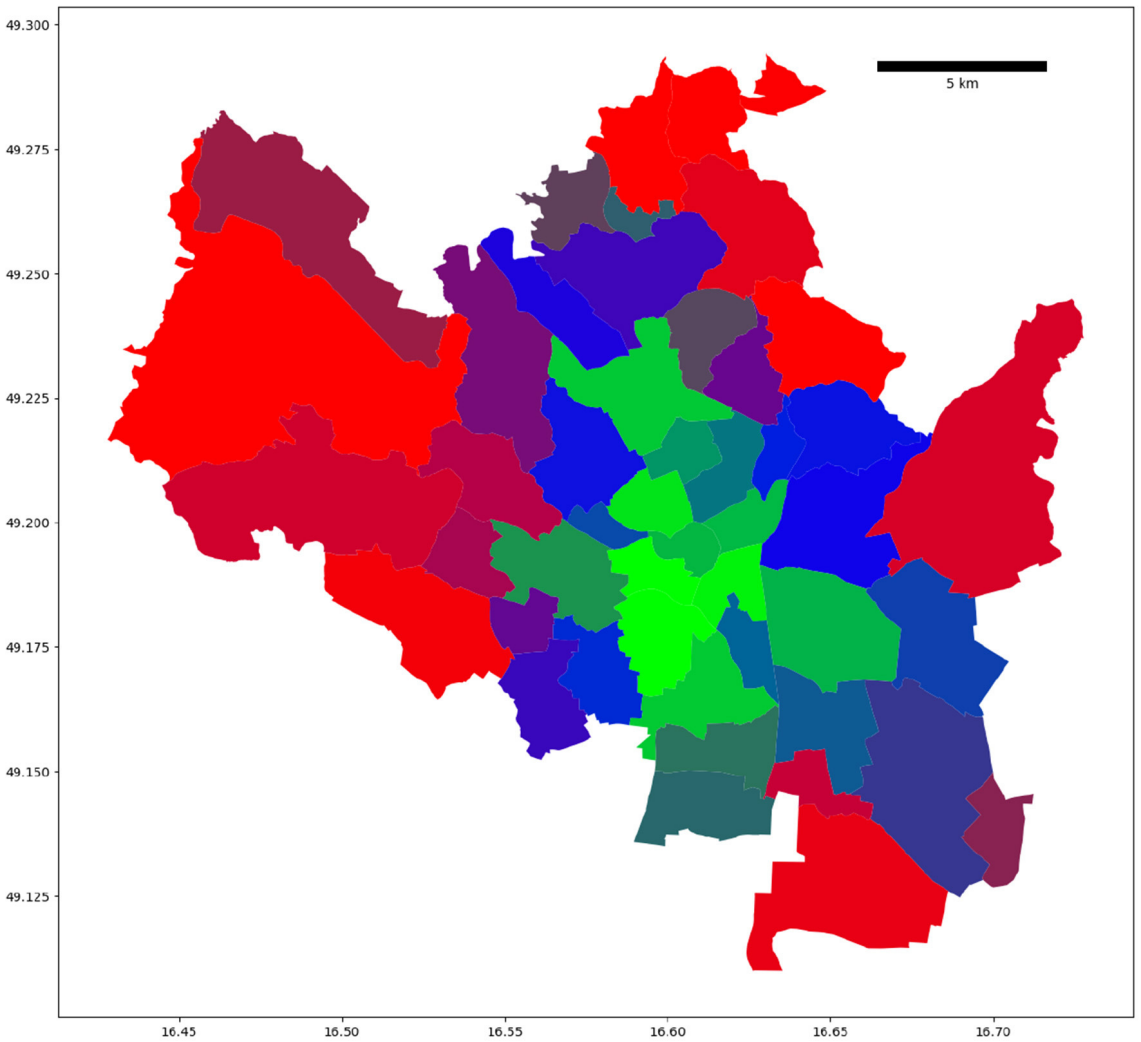
Probability-weighted clustering visualization of Brno districts. We used red to depict the residential cluster, green for the central cluster, and blue for the hybrid cluster. The district color code is computed as a linear combination of red, green, and blue colors based on clustering probabilities of the underlying clusters.

**Figure 5:**
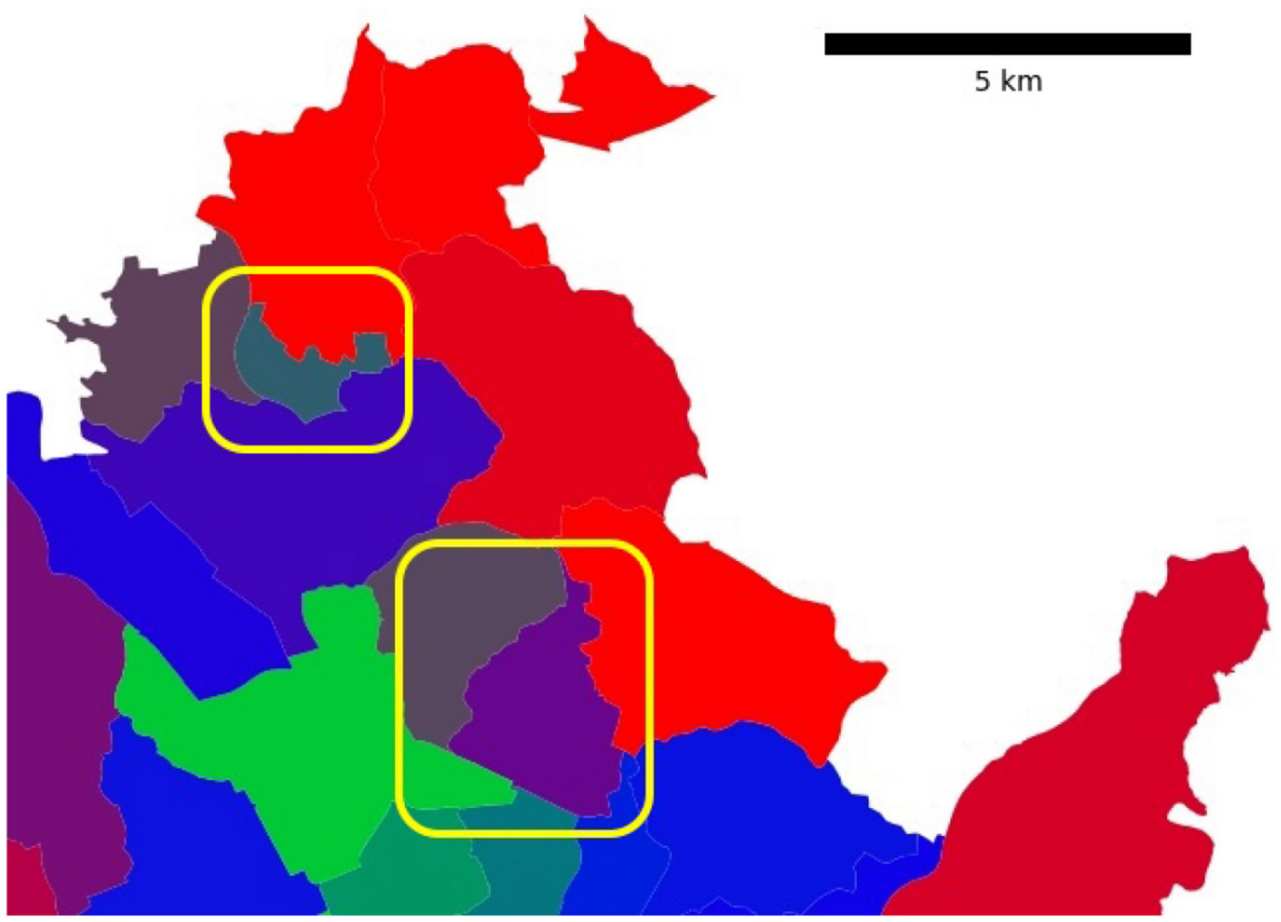
Both highlighted Brno districts belong to the hybrid cluster. The color-encoded cluster probabilities reveal traces of alternative commute patterns: the highlighted district at the top (Mokra Hora) has 43% hybrid cluster classification probability and 37% central cluster classification probability (the color is dominated by blue and green). While the highlighted district on the right side (Lesna) has 57% hybrid cluster classification probability and 41% residential cluster classification probability (the combination of blue and red results in purple).

**Figure 6:**
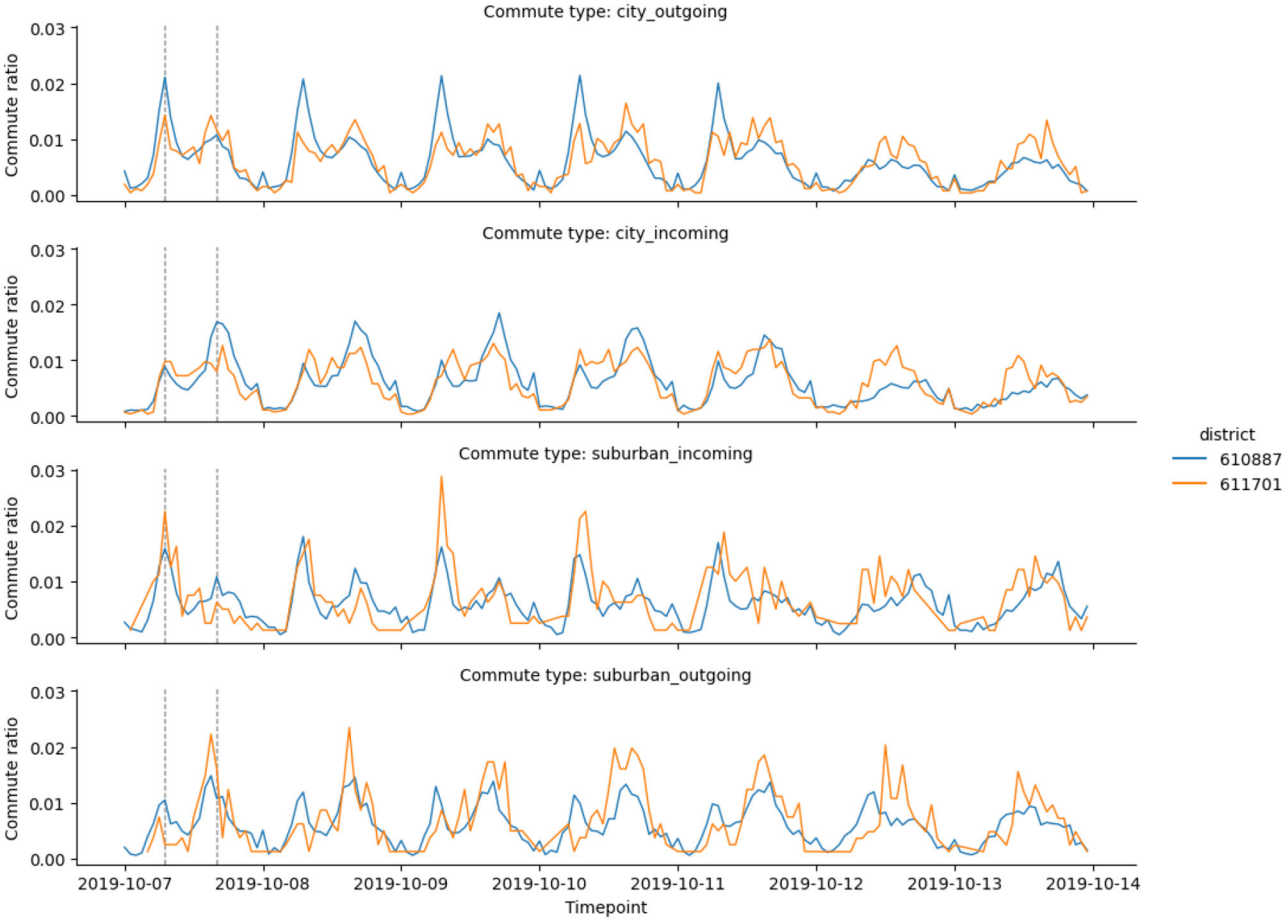
Commute timelines for the two Brno districts highlighted in Figure 5: Mokra Hora (code 611701) and Lesna (code 610887). Both districts belong to the hybrid cluster, with similar morning and evening spike patterns. Mokra Hora has a higher ratio of incoming commutes in the morning and a higher ratio of outgoing commutes in the evening. Both observations are common for central districts, attracting people in the morning.

The probability-based visualization approach (see Figure 4) reveals more information about the city commute patterns. In particular, we identified two city districts in the northern part of Brno that belong to the hybrid cluster and have different cluster probabilities (see Figure 5). To better understand the results, we compared four commute timelines for both districts (see Figure 6). The districts have similar commute patterns across all four timelines, which is expected from timelines from the same cluster. But district Mokra Hora (with code 611701) has stronger features of the central cluster, like a big spike in the suburban incoming traffic in the morning.

Mokra Hora has a 37% classification probability of the central cluster, which is an anomaly for the remote parts of the city: for comparison, the second highlighted district has only a 2.5% classification probability of the central cluster. After looking at the socioeconomic profile of the district, we found that Mokra Hora hosts one of the two largest shopping centers in the city (Kunc et al., 2012). Large shopping centers attract residents of the city and suburban areas starting in the morning and emphasize the central district commute patterns.

Overall, the combined timeline approach leads to a 6% increase in the number of central districts (compared to the previous approach). Job opportunities in European Union (EU) cities are generally concentrated in central urban areas (Smit et al., 2020), which explains why districts closer to the city center are more likely to be labeled as the central cluster when we use the suburban commute timelines, compared to the urban outflow timelines.

## 4 Discussion and conclusions

The European Parliament Resolution of December 2, 2015, on Urban Mobility [2014/2242 (INI)] contemplates the demographic forecast that by 2050, up to 82% of EU citizens will reside in urban areas other than their workplaces (Giménez-Nadal et al., 2022). The increasing ratio of suburban commuters becomes important in urban and transportation planning. In addition, the spatial and temporal distribution of suburban commute patterns may differ from the urban commute patterns. However, until recently, researchers did not have granular data to study intraday mobility patterns.

In addition to the limited availability of intraday mobility data, popular alternative sources, including smart card tracking in public transport (Ponte et al., 2021), taxi trips (Buchholz, 2015), and social-media check-in data (Wu et al., 2014) have additional challenges for suburban areas:

- Limited data coverage due to boundaries between administrative units.
- Longer commutes have an increased probability of transitions between different transportation systems.
- Suburban and residential areas attract fewer check-ins and other types of social media activities.

Our approach to studying intraday commutes is based on the passive communication between cell phones and cell towers, which makes our approach applicable to any modern city. The mobile phone data is already available to mobile phone companies, and it is a great source of data to study generic commute patterns (compared to specific datasets, like taxi or subway data). While our dataset covered only three million commute records, we explained how to scale the same approach to tens or hundreds of records.

In conclusion, the combination of urban and suburban hourly commute timelines yielded distinct and spatially cohesive clusters in Brno. These clusters revealed structural patterns delineating the city center, residential communities, and a hybrid cluster exhibiting characteristics of both. Notably, the resulting clusters displayed considerable differences in urban profiles, such as the municipal home ratio and the proportion of residents in single-family homes, varying by at least 200% among the clusters. This comprehensive approach was further validated through a quantitative comparison, demonstrating a substantial 93.75% match with a previous method while sharing only 25% of the previously considered commute timeline signature vector components. Moreover, the new clusters exhibited similar or reduced average variance values in census-based urban metrics, indicating higher district homogeneity within clusters. The probability-based visualization method provided additional insights, uncovering discrepancies within identified clusters and highlighting anomalous districts like Mokra Hora. Further analysis revealed that Mokra Hora's atypical classification probability toward the central cluster stemmed from hosting a major shopping center, influencing commuter patterns resembling those of central districts. This study's combined timeline approach led to a 6% increase in the number of districts identified as identification of central districts, aligning with the concentration of job opportunities in central urban areas within European Union cities, as previously observed in related literature (Smit et al., 2020). Understanding commute patterns is instrumental in aiding urban planners and policymakers in optimizing infrastructure development and employment distribution strategies to accommodate the diverse socio-economic characteristics and commuting patterns within urban areas.

## Data availability statement

The original contributions presented in the study are included in the article/supplementary material, further inquiries can be directed to the corresponding author.

## Author contributions

YB: Formal analysis, Investigation, Visualization, Writing—original draft. AB: Investigation, Validation, Writing—review & editing. SS: Conceptualization, Project administration, Supervision, Writing—review & editing.
